# Weed Management and Crop Establishment Methods in Rice (*Oryza sativa* L.) Influence the Soil Microbial and Enzymatic Activity in Sub-Tropical Environment

**DOI:** 10.3390/plants11081071

**Published:** 2022-04-14

**Authors:** Sarthak Pattanayak, Satyananda Jena, Priyanka Das, Sagar Maitra, Tanmoy Shankar, Subhashisa Praharaj, Prasannajit Mishra, Santanu Mohanty, Madhusmita Pradhan, Deepak Kumar Swain, Biswajit Pramanick, Ahmed Gaber, Akbar Hossain

**Affiliations:** 1 Department of Agronomy, College of Agriculture, Odisha University of Agriculture and Technology, Bhubaneswar 751003, Odisha, India; imanagronomist@gmail.com (S.P.); snjena2008@gmail.com (S.J.); prasannajitmishra71@gmail.com (P.M.); 2 Department of Agronomy, Palli Siksha Bhavana, Visva-Bharati, Sriniketan 731204, West Bengal, India; pdaspriyanka06@gmail.com; 3 Department of Agronomy, Centurion University of Technology and Management, Paralakhemundi 761211, Odisha, India; tanmoy@cutm.ac.in (T.S.); subhashisa.praharaj@cutm.ac.in (S.P.); 4 Department of Soil Science and Agriculture Chemistry, College of Agriculture, Odisha University of Agriculture and Technology, Bhubaneswar 751003, Odisha, India; santanu.madhu1960@gmail.com (S.M.); madhusmita.pradhan47@gmail.com (M.P.); 5 Department of Agricultural Statistics, Institute of Agricultural Science, Siksha-o-Anusandhan Deemed to be University, Bhubaneswar 751030, Odisha, India; deepakkswain@soa.ac.in; 6 Department of Agronomy, Dr. Rajendra Prasad Central Agricultural University, Pusa, Samastipur 848125, Bihar, India; biswajit@rpcau.ac.in; 7 Department of Biology, College of Science, Taif University, P.O. Box 11099, Taif 21944, Saudi Arabia; a.gaber@tu.edu.sa; 8 Department of Agronomy, Bangladesh Wheat and Maize Research Institute, Dinajpur 5200, Bangladesh

**Keywords:** rice, cultural methods, herbicides, impacts, soil microorganisms, soil enzymes

## Abstract

Weed management has become the most important and inevitable aspect of crop management for achieving a higher rice yield. Nowadays, chemical herbicide application has become a popular practice for managing weeds in different rice cultures. However, herbicide application can have qualitative and quantitative impacts on soil microorganisms and soil enzymes, particularly in the case of new herbicide molecules and their indiscriminate use for a longer period. Further, different rice establishment methods also play a significant role in soil microbial population dynamics as well as soil biological properties. Keeping these in view, a field experiment was conducted at the Agronomy Main Research Farm, Orissa University of Agriculture and Technology (OUAT), India, during the *kharif* season of 2016 and 2017, on the impact of crop establishment methods and weed management practices on soil microbial and enzymatic status. The field experiment was laid out in a split-plot design with three replications with four crop establishment methods in the main plot, viz., M_1_, Direct Seeded Rice (DSR); M_2_, Wet Seeded Rice (WSR); M_3_,Unpuddled Transplanted Rice (NPTR); M_4_, Puddled Transplanted Rice (PTR), and six weed management practices in the sub-plot, viz., W_1_, Weedy check; W_2_, Bensulfuron methyl 0.6% + Pretilachlor 6% (pre-emergence (PE)) 0.660 kg ha^−1^ + Hand weeding (HW) at 30 days after sowing/transplanting (days after sowing/transplanting (DAS/T)); W_3_, Bensulfuron methyl 0.6% + Pretilachlor 6% (PE) 0.495 kg ha^−1^ + HW at 30 DAS/T; W_4_, Bensulfuron methyl 0.6% + Pretilachlor 6% (PE) 0.495 kg ha^−1^ + Bispyribac-Sodium (post-emergence(POE)) 0.025 kg ha^−1^ at 15 DAS/T; W_5_, Cono weeding (CW) at 15 DAS/T + hand weeding 30 DAS/T, and W_6_, Brown manuring/Green manuring. The initial decline in the microbial population was observed due to herbicide application in NPTR and PTR up to 7 DAS/T and then it increased up to 28 DAS/T. There was a reduction in soil microbial and enzymatic status after the application of herbicides Bensulfuron methyl 0.6% + Pretilachlor 6% (PE) and Bispyribac-Sodium (POE) that again followed an upward graph with crop age. Significant variation in enzymatic activity and the microbial count was also observed among treatments involving crop establishment methods. The study revealed that improved microbial population and enzyme activity were noted in unpuddled transplanted rice under organic weed management due to favorable conditions, and chemical weed control initially affected microbial population and activities.

## 1. Introduction

Rice (*Oryza sativa* L.) is the most widely consumed staple food for more than 50% of the world’s human population. Around 90% of the global rice production is being grown and consumed in Asia where rice is synonymous with the livelihood of people [1,2]. Globally, rice is grown in 120 countries in an area of 164.1 million hectares with a production of 756.74 million tons and productivity of 4.6 t ha^−1^ [3]. India is the second-largest rice-producing country in the world where rice is the main staple food crop. In India, rice occupies 4.4 million hectares with a production of 118 million tons and productivity of 2.7 t ha^−1^ [4]. Odisha, one of the leading rice-growing states of India, grows rice in 3.89 Mha with a production of 8.04 million tons and productivity of 2.1 t ha^−1^, which is low compared to global and national average productivity [4].

Achieving self-sufficiency in rice production and maintaining price stability are important objectives in low-income countries [5]. Kharif rice is grown in different ecosystems in Odisha such as rainfed upland, rainfed lowland, deep water, and tidal wetland thanks to the high adaptability of rice to a wide range of geohydrological situations. Challenges to rice cultivation have increased manyfold due to the impact of climate change, shrinking landmass, labor, and water scarcity. This has triggered the need to revisit crop management strategies, more importantly, crop establishment methods, and to formulate better weed management strategies for higher rice production.

Life and biodiversity of soil may be prone to destruction due to the intensive use of plant protection chemicals [6]. Plant protection materials are chemically derived compounds containing one or more than one active ingredients with mutually complementary properties but with different modes of action [7] and their application is exceptionally beneficial for agriculture. However, we have to realize the threats attached to soil microbial diversity [8,9,10]. The application of plant protection chemicals exceeding the recommended dose may impact the growth and development of microbial assemblages [11], plants [12], animals [13], and people [14]. It has been experimentally demonstrated by many researchers that only a small percentage of applied plant protection chemicals are actually involved in controlling target organisms while a huge percentage pervades soil, water, and living organisms [15,16].

Climatic condition with soil physicochemical properties affects the persistence of these chemicals in soil and their movement to water and agroecosystem [17]. Various effects of plant protection chemicals can be seen on soil microorganisms as well as detrimental impacts on some microorganisms [18,19]. Microorganisms are an excellent source of carbon and energy and so they play multifaceted roles in the agroecosystem inclusive of enhancement of plant nutrients and bioremediation of soil from harmful abiotic factors [17,20,21]. Herbicide application results in better control of weeds with higher weed control efficiency and a lower labor requirement. Initially, herbicide application was mostly confined to plantation crops, but nowadays it is mostly used in field crops where wheat and rice constitute around 42% and 32% of total herbicide consumption, respectively [22].

However, herbicide application can have a qualitative and quantitative impact on the alternation of soil microbial population and soil enzymatic activity [23]. Soil microorganisms and plant roots are an important source of soil enzymes, therefore, the effect of herbicides on soil microorganism invariably affect soil enzymatic activity [16,18]. Moreover, a change in the soil microflora has been listed as one of the possible causes of productivity decline in rice-based cropping systems [24]. Soil microbial biomass is considered an active nutrient supply pool to plants. Herbicides are considered to have no major or long-term effect on soil microbial counts when applied at the standard field recommended dose. However, indiscriminate use of herbicides and new molecules of herbicide may affect soil health, particularly soil microorganisms and soil enzymatic activity, and may lead to long term accumulation.

The Bensulfuron methyl, methyl ester of bensulfuron, an acetolactase synthatse inhibitor and pretilachlor [2–chloro–2,6–diethyl–N–(2–propoxyethyl) acetanilide] is a chloroacetanilide herbicide. Bensulfuron methyl + pretilachlor is a new herbicide combination reported to be used as a pre-emergence herbicide providing effective control of grasses, sedges, and broad-leaved weeds in rice without any phytotoxic symptoms in the crop [25]. Bispyribac sodium is popularly used as a post-emergence herbicide in rice [26,27], it acts on the enzyme acetolactase synthetase (ALS), which in turn inhibits the production of amino acids such as valine, isoleucine, and leucine [28] that inhibit protein synthesis in plants, causing their death. Due to the rapid photochemical transformation and low vitalization potential, its environmental impact is considered to be negligible and is moderate to highly persistent in anaerobic flooded rice soil [29]. Detailed studies on the impact of new molecules are lacking, demanding an experiment to establish the cause and effect. 

On the other hand, brown and green manuring practices are effective in managing the weed population in rice and improving soil health [30,31]. Knowledge of the effect of herbicides on soil biological activity and crop yield is very helpful in developing a better crop management strategy. Further, rice establishment methods have a significant impact on the growth and productivity of crops inclusive of microbial activities [32,33]. Variation in water regime and associated cultural operations may result in varying microbial count and enzymatic activity. The variation in microbial activity was earlier recorded under different rice establishment methods by Latha and Gopal [34] and Ramalakshmi et al. [35]. 

Considering the above facts, this study was planned to evaluate microbial population dynamics, namely, total bacteria, fungi, and actinomycetes, and soil enzymatic activity of soil enzymes (viz., urease, alkaline phosphatase, and dehydrogenase) in *Kharif* rice under various weed management and crop establishment methods. This will help to evaluate the effectiveness of new herbicide molecules along with the impact of different weed management practices through organic and mechanical methods on soil health and soil biological properties, and will also enable researchers, policy makers, and farmers to evaluate better treatment combinations for higher yield by maintaining soil health.

## 2. Materials and Methods

### 2.1. Site Description

The experiment was conducted during two Kharif seasons in 2016 and 2017 at the Agronomy Main Research Farm, Department of Agronomy, Odisha University of Agriculture and Technology, Bhubaneswar, Odisha, India which lies at 20°15′N latitude and 85°52′E longitude, respectively, with an altitude 25.9 m above the mean sea level. The station falls under the Agro-Climatic Zone of the East and Southeastern Plain Zone of Odisha, India with a moisture deficit index (MDI) of −0 to −20 and a growing season length of 180 to 210 days. The climate of the area is warm and moist with hot humid summer and mild winter. Broadly, the climate falls in the category of moist hot [36]. The Rainfall code of Bhubaneswar, Odisha, India is D_1_A_2_ (B_1_A_2_B_1_) C_1_D_1_E_2_.

Representative soil samples were collected from a depth of 0–10 cm in a zig-zag manner using a soil auger before the beginning of the experiment to analyze and study the soil textural classes and soil physiochemical and biological properties; each sample was a composite of three locations within a plot. The experimental soil was slightly acidic and sandy loam in texture with a medium organic matter content with low soil available nitrogen, high phosphorus, and medium available potassium. The data on soil textural class and soil chemical and biological characteristics are given in Table 1, Table 2 and Table 3.

### 2.2. Meteorological Conditions

**Figure 1:**
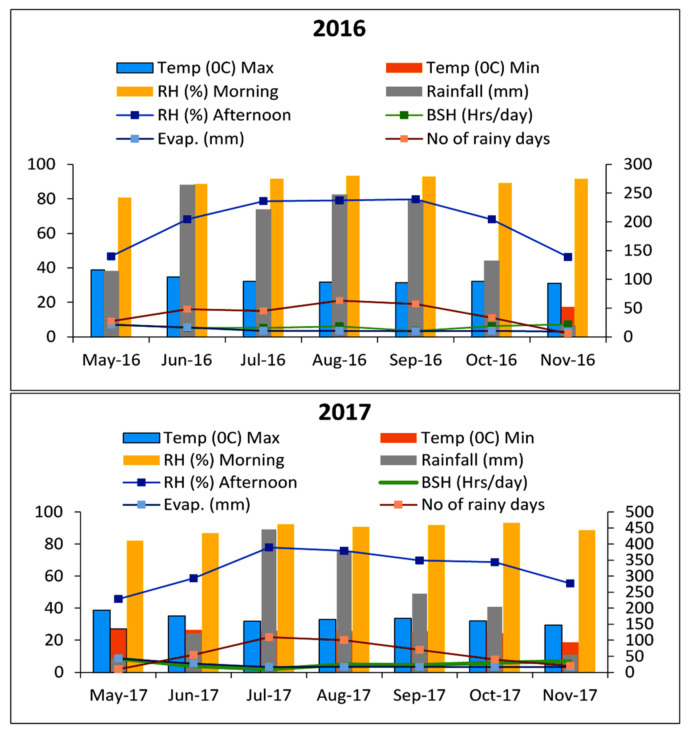
Monthly meteorological data during crop growing season (*kharif*) during 2016 and 2017 (Department of Agrometeorology, College of Agriculture, OUAT, Bhubaneswar, Odisha, India).

Detailed weather parameters of the growing seasons of both years are given in Figure 1. 

The maximum temperature was recorded in May (38.8 °C) followed by 34.8 °C in June, while the lowest of 17.4 °C was in November 2016. Similarly, the maximum temperature of 38.8 °C was recorded in May followed by 35.2 °C in June while November recorded the lowest at 18.7 °C in the year 2017. Rainfall received from May to November in 2016 and 2017 was 1241 mm (93 rainy days) and 1492.9 mm (81 rainy days), respectively (Department of Agrometeorology, College of Agriculture, OUAT, Bhubaneswar, Odisha, India). 

### 2.3. Treatment and Layout

The field experiment was laid out in a split-plot design with a standard gross plot size of 5.8 m × 4.5 m with three replications by taking twenty-four treatment combinations with four crop establishment methods in the main plot and six weed management practices in the sub plots (Table 4).

### 2.4. Crop Culture

Medium (115–130 days) duration rice variety Naveen (parentage- CR-749-20-2/IET-14461) with medium bold variety was taken as the test crop.

#### 2.4.1. Rice Establishment Methods

As stated earlier, the following are the details of the crop establishment methods:

Direct Seeding (M_1_): Seeds were directly sown in solid rows at 20 cm row-to-row spacing on well-pulverized soil.

Wet Seeding (M_2_): The seeds were soaked for 24 h in water and were incubated for 48 h for sprouting; sprouted seeds were directly sown in solid rows at a spacing of 20 cm row-to-row on puddled soil.

Unpuddled Transplanted Rice (M3): 21 days old seedlings raised in a dry nursery were transplanted at two seedlings per hill at a spacing of 20 cm × 10 cm in well-cultivated non-puddled soil. Irrigation was applied to moisten the soil and allowed to settle for 12–24 h before transplanting in unpuddled transplanted treatment.

Puddled Transplanted Rice (M4): 21 days old seedlings raised in a dry nursery were transplanted at two seedlings/hill at a spacing of 20 cm × 10 cm in puddled transplanted soil.

Well decomposed Farm Yard Manure (FYM) at the rate of 5 t ha^−1^ was incorporated into the soil at final land preparation and inorganic fertilizers at 80-40-40 kg ha^−1^ N, P_2_O_5_, K_2_O were applied to all the plots irrespective of treatments imposed. The full dose of P and K and 25% of N were applied at final plowing/puddling as basal dose. However, the rest of N was applied in a 2:1 ratio at tillering and panicle initiation stages, respectively, through Urea (46-0-0). The fertilizers used in the study were urea, diammonium phosphate (18-46-0), and muriate of potash (0-0-60).

#### 2.4.2. Weed Management Practices

The application of the treatments related to weed management practices tested in the study has been described below:

Brown manuring/Green manuring: Sesbania seed was sown at 25 kg ha^−1^ together with rice. After 25–30 days of growth, when Sesbania was 30–40 cm tall, it was killed with 2, 4-D ester at 0.5 kg ha^−1^.

Cono weeding and hand weeding: Cono weeding with a cono weeder, a mechanical farm implement, was operated in the inter-row at 15 DAS/T and hand weeding was operated with manual hand weeder at 30 DAS/T in W_5_ plots while hand weeding operation in W_2_ and W_3_ plots was carried out at 30 DAS/T.

Herbicide application: Granular herbicides were uniformly applied to plots as per treatments after mixing with sand while liquid herbicide was applied by a knapsack sprayer using a flat fan nozzle as per treatments so as to spray the fluid uniformly throughout the targeted area. Bensulfuron Methyl (0.6%) + Pretilachlor (6%) (Ready-mix) was applied as pre-emergence herbicide 3 days after sowing/transplanting (DAS/T) at two doses at 0.660 kg ha^−1^ (W_2_) and 0.495 kg ha^−1^ (W_3_) while Bispyribac-Sodium (POE) 0.025 kg ha^−1^ was applied as post-emergence herbicide at 15 DAS/T in (W_3_). For further details, refer to Table 3.

### 2.5. Microbial Analysis

#### 2.5.1. Enumeration of Soil Microbial Population

Serial dilution and spread plate techniques were adopted to determine the soil microbial population, where one gram of the soil samples was added to ten tubes containing 9 ml of distilled water, serially diluted, and spread over nutrient agar and potato dextrose agar for enumeration of total bacteria and actinomycetes and fungi, respectively. The plates were incubated at 30 °C for 24 h for bacterial isolation and 48 h for actinomycetes and fungi. The following formula was considered for the calculation of soil microbial population.

(1) $$\mathrm{CFU}/\mathrm{mL}=\frac{\mathrm{No}.\;\mathrm{of}\;\mathrm{colony}\;\times \;\mathrm{inverse}\;\mathrm{of}\;\mathrm{dilution}\;\mathrm{taken}}{\mathrm{Volume}\;\mathrm{of}\;\mathrm{inoculum}\;\mathrm{taken}}$$

Here, CFU denoted the colony-forming unit of microbes, and ml represented milliliter.

#### 2.5.2. Urease Activity

The method adopted to evaluate the urease activity of soil was essentially the same as adopted by Pancholy and Rice [43], except that the ammonia liberated due to hydrolysis used in the reaction mixture was determined by nesslerization as described by Jackson [38]. Ten grams of each freshly collected soil sample was placed in 100 mL capacity Erlenmeyer flasks to which 1 mL toluene was added and allowed to stand for 15 min to permit complete penetration into soils. Each of these flasks then had 10 mL of phosphate buffer (pH 6.7) and 10 mL of 10% urea solution added. For control flasks, the urea solution was replaced by an equal quantity of distilled water. The contents of the flasks were well shaken for 5 min and incubated at 30 °C for 24 h. The contents of the flask were filtrated through Whatman No. 42-filter paper after incubation. The remaining soil in the flask was mixed with 15 mL of 1 N KCl solution, shaken for 5 min, and filtered. The volume of the total filtrate was increased to 100 mL in a volumetric flask using distilled water. 

The ammonia present in the filtrate was determined by nesslerization. A total of 1 mL filtrate of each sample was transferred to a 20 mL volumetric flask to which 2 mL tartrate solution and 0.5 mL Nessler’s reagent were added. The volume was increased to 20 mL with distilled water. The yellow color developed after 30 min was measured at 410 nm using a Graphicord Shimadzu UV-visible spectrophotometer (model UV 240) against the reagent blank.

#### 2.5.3. Soil Phosphatase (Acid and Alkaline) Activity

Soil phosphatase activity involved colorimetric estimation of the p-nitrophenol released by phosphatase activity when soil is incubated with buffered (pH 6·5 and 11) sodium p-nitrophenyl phosphate solution and toluene at 37 °C for 1 h [42]. First, 1 g of fresh soil was weighed. To it, 0.2 mL of toluene was added. Then, 4 mL acid phosphatase MUB buffer (pH 6.5 or pH 11) was added, respectively, for acid phosphatase or alkaline phosphatase. Then, 1 mL of p-nitrophenol phosphate solution was mixed and placed in an incubator at 37 °C for 1 h. After 1 h, 1 mL of 0.5(M) CaCl_2_ and 4 mL of 0.5(M) NaOH was added and shaken for 1 min. The soil suspension was filtered. The yellow color intensity was measured at 420 nm by spectrophotometer.

#### 2.5.4. Soil Dehydrogenase Activity

The soil dehydrogenase activity was measured following the method of reduction of 2, 3, 5- triphenyl tetrazolium chloride (TTC) to the creaming red-colored triphenyl formazan (TPF). First, 3 g of fresh soil was weighed. To it, 0.03 g CaCO_3_ and 1 mL of TTC were added. Then, 2.5 mL of distilled water was mixed and placed in an incubator at 37 °C for 24 h. After 24 h, 10 mL methanol was added and shaken for 1 min. The soil suspension was filtered. The filtered solution volume was increased to 100 mL with methanol. The red color intensity was measured at 485 nm by spectrophotometer [41,44].

### 2.6. Statistical Analysis

The experimental data were analyzed by adopting Fisher’s method of analysis of variance as outlined by Gomez and Gomez [45] to draw a valid conclusion. The variations in the treatment mean were tested by using critical difference (CD) values at a 5% level of significance. The ANOVA model adopted for the above experiment was as follows:

$$Y_{ijk}=\mu +\rho_{i}+\tau_{j}+\delta_{ij}+\beta_{k}+{\left(\tau \beta \right)}_{jk}+\varepsilon_{ijk}$$

where i = 1, 2, …, r, j = 1, 2, …, a, k = 1, 2, …, b;$\;Y_{ijk}\;$= observation corresponding to the *k*th level of sub-plot factor(W), *j*th level of main plot factor (M), and ith replication. μ = general mean, $\rho_{i}=$ *i*th block effect,$\;\tau_{j}\;$= *j*th main plot effect $\beta_{k}\;$= *k*th sub-plot effect,$\;{\left(\tau \beta \right)}_{jk}\;$= interaction between the *j*th level of main plot treatment and the *k*th level of sub-plot treatment.

The error components $\delta_{ij}$ and $\varepsilon_{ijk}$ are independently and normally distributed with means zero and respective variances $\sigma_{\delta }^{2}$ $\mathrm{and}\;\sigma_{\varepsilon }^{2}$.

Correlation and coefficient analysis was made as per the statistical methods (Cochran and Cox [46].

## 3. Results

### 3.1. Effect of Crop Establishment Methods and Weed Management Practices on Soil Microbial Population

The total bacterial, fungal, and actinomycetes population was significantly influenced by the type of weed management practices, types of herbicides, their concentration, and the days after herbicide application.

#### 3.1.1. Effect of Crop Establishment Methods and Weed Management Practices on Total Bacterial Population

Crop establishment methods and weed management practices in rice significantly influenced the total bacterial population and data on different days after sowing/transplanting are presented in Table 5. 

A declining trend was seen in the total bacterial population at 7 DAS/T in all establishment methods from the initial total bacterial population of 41.9 × 10^6^ CFU/g soil. Among all establishment methods, there was a higher decline in the bacterial population in PTR (35.93 and 37.78), followed by WSR (38.53 and 39.38) and DSR (37.57 and 38.48) in both years, respectively. While the lowest decline in the total bacterial population at 7 DAS/T was recorded in NPTR treatment (39.47 and 40.50, respectively) during both years. Thereafter, a continuous increase in the bacterial population was seen at 14 DAS/T, 21 DAS/T, and 28 DAS/T. Among the four different establishment methods taken, significantly higher values of the bacterial population were recorded in unpuddled transplanted rice (NPTR) viz. at 14 DAST/T (43.82 and 44.18), 21 DAST/T (45.67 and 45.83), and 28 DAS/T (49.57 and 48.85) during both years of the experiment, respectively, which was followed by wet seeded rice (WSR) viz. 14 DAS/T (41.87 and 43.33), 21 DAS/T (44.08 and 44.70), and at 28 DAS/T (45.33 and 45.47), respectively, during both years (Table 5).

Among the six different weed management strategies taken, the total bacterial population increased in brown/green manuring, weedy check, and CW+HW treatments where no chemicals were applied, while the total bacterial population declined in treatments where chemical herbicides were applied viz., Bensulfuron methyl 0.6% + Pretilachlor 6% at 0.495 kg ha^−1^ and Bensulfuron methyl 0.6% + Pretilachlor 6% (PE) at 0.660 kg ha^−1^ at initial 7 DAS/T. Thereafter, there was a continuous increase in total bacterial population in all treatments until 28 DAS/T, except for the treatment Bensulfuron methyl 0.6% + Pretilachlor 6% (PE) at 0.495 kg ha^−1^ + Bispyribac-Sodium at 0.025 kg ha^−1^ POE where there was a decline at 21 DAS/T (33.98 732.55) from 14 DAS/T (35.83 and 39.40) which may be due to Post-emergence application of Bispyribac-Sodium at 0.025 kg ha^−1^ at 15 DAS/T, but the bacterial population also increased in same treatment at 28 DAS/T (38.43 and 41.45, respectively) during both years. Bensulfuron methyl 0.6% + Pretilachlor 6% (PE) at 0.660 kg ha^−1^ and Bensulfuron methyl 0.6% + Pretilachlor 6% (PE) at 0.495 kg ha^−1^ recorded a 22.67% and 18.13% decrease, respectively, in total bacterial population over the initial value at 7 days after sowing, 3 days after herbicide application.

Application of Bensulfuron methyl 0.6% + Pretilachlor 6% (PE) at 0.660 kg ha^−1^ + HW at 30 DAS/T, Bensulfuron methyl 0.6% + Pretilachlor 6% (PE) at 0.495 kg ha^−1^ + HW at 30 DAS/T and Bensulfuron methyl 0.6% + Pretilachlor 6% (PE) at 0.495 kg ha^−1^ + Bispyribac-Sodium at 0.025 kg ha^−1^ POE reduced the total bacterial population by 1.8%, 7.6%, and 25.8% while brown/green manuring resulted in a 16.2% increase over weedy check at 21 DAS/T.

#### 3.1.2. Effect of Crop Establishment Methods and Weed Management Practices on Total Fungi Population

The reduction in total fungal population from the initial values of 11.9 (×10^4^ CFU g^−1^ soil) was seen at 7 DAS/T in all establishment methods taken, while the highest decline was seen in Direct seeded rice (9.95 and11.43) which was significantly at par with the fungal population of puddled transplanted rice (9.87 and11.62, respectively) during both years (Table 6).

The lowest decline was recorded in unpuddled transplanted rice (11.28) during the first year while the lowest decline in the second year was recorded in Wet seeded rice (12.48). Thereafter, a constant increase in the total fungal population was seen during the stage of data recording. Significantly, higher values of the total fungal population were recorded in NPTR at 14 DAS/T (17.35 and 21.47), 21 DAS/T (20.80 and 23.47), and 28 DAS/T (28.92 and 31.98, respectively) during both years among all other crop establishment methods taken (Table 6).

Brown manuring/green manuring, CW + HW, and weedy check recorded a constant increase in total fungal population, while Bensulfuron methyl 0.6% + Pretilachlor 6% (PE) at 0. 660 kg ha^−1^ and Bensulfuron methyl 0.6% + Pretilachlor 6% (PE) at 0.495 kg ha^−1^ recorded a 31.93% and 20.12% decrease in total fungal population over the initial value at 7 days after sowing, 3 days after herbicide application. Brown/green manuring treatment recorded a higher total fungal population at 7 DAS/T (12.65 and 15.45), 14 DAS/T (19.68 and 21.85), and 21 DAS/T (24.78 and 27.65, respectively). CW + HW recorded higher values of total fungal population (28.00 and 30.40) which was significantly at par with treatment with Bensulfuron methyl 0.6% + Pretilachlor 6% (PE) at 0.660 kg ha^−1^ + HW at 30 DAS/T (27.75 and 30.63) and was followed by the application of Bensulfuron methyl 0.6% + Pretilachlor 6% (PE) at 0.495 kg ha^−1^ + HW at 30 DAS/T (26.88 and 29.70) at 28 DAS/T, respectively, during both years (Table 6).

#### 3.1.3. Effect of Crop Establishment Methods and Weed Management Practices on Total Actinomycetes Population

The data recorded on the total count of actinomycetes recorded at various growth stages are presented in Table 6. Apart from the unpuddled transplanted rice, where there was a slight increase in total actinomycetes population (34.83 and 34.38), there was a decline in total actinomycetes population in all crop establishment methods at 7 DAS/T. Significantly higher values of total actinomycetes were seen in NPTR at 14 DAS/T (39.55 and 39.15), 21 DAS/T (42.65 and 42.45), 28 DAS/T (48.22 and 45.23, respectively) during both years (Table 7).

Brown/green manuring recorded higher values of total actinomycetes population from 7 DAS/T (40.58 and 39.18), 14 DAS/T (44.63 and 43.53), 21 DAS/T (48.08 and 47.35), while CW+ HW recorded higher values at 28 DAS/T (50.00 and 49.35, respectively) during both years. There was a continuous increase in total actinomycetes population in all weed management practices from 7 DAS/T to 28 DAS/T apart from the treatment of Bensulfuron methyl 0.6% + Pretilachlor 6% (PE) at 0.495 kg ha^−1^ + Bispyribac-Sodium at 0.025 kg ha^−1^ POE (W_4_) where there was a decline in total actinomycetes population at 21 DAS/T (24.60 and 29.33) from 14 DAS/T (31.90 and 33.58) that again increased at 28 DAS/T (35.88 and39.53) during both test years, respectively. Bensulfuron methyl 0.6% + Pretilachlor 6% (PE) at 0.495 kg ha^−1^ + Bispyribac-Sodium at 0.025 kg ha^−1^ POE treatment recorded 36.01% less actinomycetes population than weedy check at 21 DAS/T. 

### 3.2. Effect of Crop Establishment Methods and Weed Management Practices on Soil Enzymatic Activities

#### 3.2.1. Effect of Crop Establishment Methods and Weed Management Practices on Urease Activity

Data on urease activity in rice soil was significantly affected by crop establishment methods and weed management practices and is presented in Table 8. Brown/green manuring recorded higher values of total actinomycetes population from 7 DAS/T (40.58 and 39.18), 14 DAS/T (44.63 and 43.53), 21 DAS/T (48.08 and 47.35), while CW+ HW recorded higher values at 28 DAS/T (50.00 and 49.35, respectively) during both years. There was a continuous increase in total actinomycetes population in all weed management practices from 7 DAS/T to 28 DAS/T apart from the treatment of Bensulfuron methyl 0.6% + Pretilachlor 6% (PE) at 0.495 kg ha^−1^ + Bispyribac-Sodium at 0.025 kg ha^−1^ POE (W_4_) where there was a decline in total actinomycetes population at 21 DAS/T (24.60 and 29.33) from 14 DAS/T (31.90 and 33.58) that again increased at 28 DAS/T (35.88 and39.53) during both test years, respectively. Bensulfuron methyl 0.6% + Pretilachlor 6% (PE) at 0.495 kg ha^−1^ + Bispyribac-Sodium at 0.025 kg ha^−1^ POE treatment recorded 36.01% less actinomycetes population than weedy check at 21 DAS/T. Urease activity declined at 7 DAS/T from initial values and increased thereafter with days after sowing/transplanting. PTR recorded the highest urease activity at all stages of data recorded viz. 7 DAS/T (24.57 and 27.00), 14 DAS/T (26.57 and 30.58), 21 DAS/T (27.44 and 31.36), and 28 DAS/T (30.53 and 35.02). At 7 DAS/T, the trend of urease activity was PTR > WSR > NPTR > DSR and at 28 DAS/T the trend was PTR > WSR> DSR > NPTR. PTR, WSR, NPTR, and DSR recorded a 3.4%, 7.9%, 10.1%, and 15.4% decline in urease activity from the initial value of 26.7 μg NH3 released g^−1^ soil h^−1^ at 7 DAS/T. Bensulfuron methyl 0.6% + Pretilachlor 6% (PE) at 0.660 kg ha^−1^, Bensulfuron methyl 0.6% + Pretilachlor 6% (PE) at 0.495 kg ha^−1^ recorded a 28.1% and 25.1% decrease in urease activity than the initial value, while weedy check, CW+HW, and brown/green manuring resulted a 7.05%, 7.0%, and 7.3% higher urease activity than initial values at 7 DAS/T. Brown/green manuring recorded higher urease activity at all stages of data recording, 7 DAS/T (27.15 and 30.50), 14 DAS/T (28.80 and 32.60), 21 DAS/T (33.28 and 34.49), and 28 DAS/T (33.30 and 34.10) (Table 8).

Among the different chemicals, the herbicide application of Bensulfuron methyl 0.6% + Pretilachlor 6% (PE) at 0.660 kg ha^−1^ recorded a higher decline in urease activity than the application of Bensulfuron methyl 0.6% + Pretilachlor 6% (PE) at 0.495 kg ha^−1^ at 7 DAS/T. There was a continuous increase in urease activity while in treatment with the application of Bensulfuron methyl 0.6% + Pretilachlor 6% (PE) at 0.495 kg ha^−1^ + Bispyribac-Sodium at 0.025 kg ha^−1^ POE (W4) and there was a decline in urease activity at 21 DAS/T (17.92 and 22.23) from 7 DAS/T (23.15 and 24.30); this may be due to post-emergence application of Bispyribac-Sodium at 0.025 kg ha^−1^ (23.15 and 24.30) which increased thereafter at 28 DAS/T during both years of experiment.

#### 3.2.2. Crop Establishment Methods and Weed Management Practices Influence the Alkaline Phosphatase Activity

Data on alkaline phosphatase activity in rice soil was significantly affected by crop establishment methods and weed management practices and is presented in Table 9. A perusal of data revealed that values of alkaline phosphatase declined at 7 DAS/T, irrespective of crop establishment methods from the initial values; however, the decline was lowest in unpuddled transplanted rice, followed by WSR. Thereafter, the values of alkaline phosphatase increased with the age of crop production. Significantly higher values of alkaline phosphatase were recorded in NPTR at all the stages of data recording viz., 7 DAS/T (171.32 and 175.68), 14 DAS/T (186.28 and 191.70), 21 DAS/T (192.17 and 197.42), and at 28 DAS/T (197.95 and 215.47), followed by WSR viz. 7 DAS/T (163.05 and 169.05), 14 DAS/T (179.93 and 179.53), 21 DAS/T (185.90 and 188.55), and 28 DAS/T (190.45 and 201.07, respectively) during both years. Alkaline phosphatase values declined after herbicide application or a higher concentration of herbicide applied, resulting in an additional decline in enzymatic activity. After 7 DAS/T, the alkaline phosphatase value was higher in brown/green manuring followed by CW + HW and weedy check.

There was an initial decline in values of alkaline phosphatase in treatments with chemical herbicide application. In W_2_ treatment, the application of Bensulfuron methyl 0.6% + Pretilachlor 6% (PE) at 0.660 kg ha^−1^ had an initial decline of alkaline phosphatase values (141.83 and 146.58), while in W_3_ and W_4_, the values were 148.70 and 152.70, respectively, during both years. However, higher values were recorded in W_1_, W_5,_ and W_6_. After the initial decline, values of alkaline phosphatase increased with days of crop production, except in treatment W_4_ (Bensulfuron methyl 0.6% + Pretilachlor 6% (PE) at 0.495 kg ha^−1^ + Bispyribac-Sodium at 0.025 kg ha^−1^ POE) at 21 (149.10 and 149.05) from 14 DAS/T (160.03 and 158.93), which may be due to the post-emergence application of Bispyribac-Sodium at 0.025 kg ha^−1^ at 15 DAS/T, while the values again increased at 28 DAS/T (178.65 and 181.33), during both years, respectively.

#### 3.2.3. Crop Establishment Methods and Weed Management Practices Influence the Dehydrogenase Activity

Data on dehydrogenase activity in rice soil was significantly affected by crop establishment methods and weed management practices and is presented in Table 10. From the study, it is revealed that there was a decline in soil enzymatic activity at 7 DAS/T, which is 4 days after application of pre-emergence herbicide in all crop establishment methods, apart from unpuddled transplanted rice, where a slight increase in dehydrogenase activity was recorded. Thereafter, from 7 DAS/T, soil enzyme dehydrogenase activity increased with crop age. The non-puddled transplanted rice (NPTR) condition recorded the highest dehydrogenase enzyme activity at all stages of data recording, viz. 7 DAS/T (84.43 and 86.07), 14 DAS/T (94.32 and 96.03), 21 DAS/T (98.92 and 107.43), and 28 DAS/T (103.65 and 112.95, respectively) during both years. Among the different chemical weed management practices adopted, the highest decline of initial dehydrogenase activity was recorded with the application of Bensulfuron methyl 0.6% + Pretilachlor 6% (PE) at 0.660 kg ha^−1^ (72.55 and 71.95), followed by the application of Bensulfuron methyl 0.6% + Pretilachlor 6% (PE) at 0.495 kg ha^−1^ (74.28 and 74.73, respectively) during both years (Table 10).

Among all the weed management practices adopted, higher dehydrogenase activity was recorded in brown/green manuring (97.53 and 98.00), which was followed by CW + HW (86.63 and 88.65) and weedy check (86.63 and 88.20, respectively) during both years. Thereafter, an increase in soil enzymatic activity was recorded with crop age in all treatments of weed management applied, apart from the treatment with application of Bensulfuron methyl 0.6% + Pretilachlor 6% (PE) at 0.495 kg ha^−1^ + Bispyribac-Sodium at 0.025 kg ha^−1^ POE, at 21 DAS/T (75.48 and 75.00) that again increased at 28 DAS/T (92.30 and 90.48, respectively) during both years. 

### 3.3. Correlation between Micro-Organisms and Soil Enzymes at Different Growth Stages 

Multiple correlations between micro-organisms and soil enzymes (*n* = 24) at different stages recorded at 7, 14, 21, and 28 DAS in both years are presented in Table 11 and Table 12. Data in both Tables revealed that there was a positive significant correlation between micro-organisms and soil enzymes recorded at 7, 14, 21, and 28 DAS/T in both years.

### 3.4. Effect of Crop Establishment Methods and Weed Management Practices on Species Wise Weed Count

**Figure 2:**
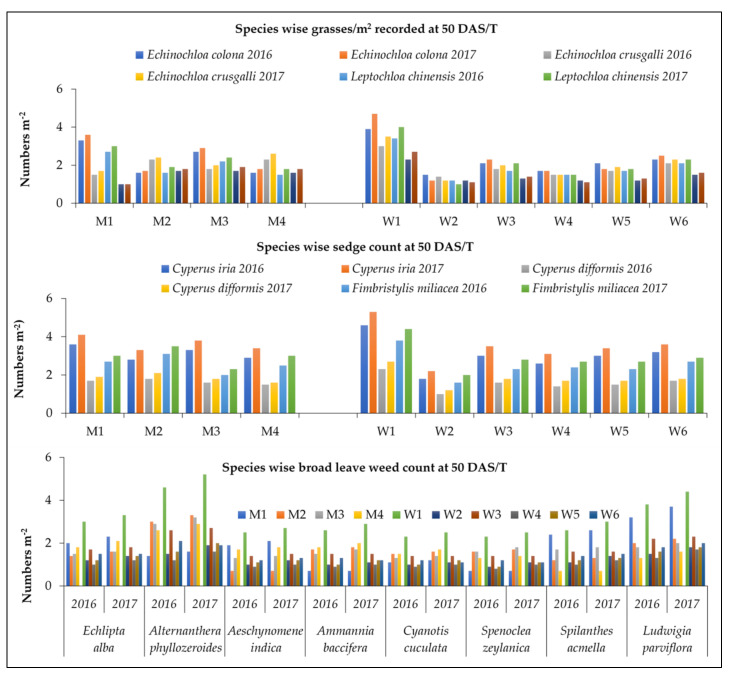
Effect of crop establishment methods and weed management practices on species-wise weed count at 50 DAS/T.

Among grasses, *E. colona* and *Leptochloa chinensis* were prominent in DSR, while *E. crusgall* and *Paspalum distichum* was prominent in WSR and PTR (Figure 2). Among sedges, *Cyperus iria* was prominent in DSR while *C. difformis* and *Fimbristylis miliacea* were prominent in WSR. Among BLWs, *Eclipta alba, Ludwigia parviflora,* and *Spilanthes acmella* were prominent in DSR, *Aeschynomene indica, Alternanthera philoxeroids,* and *Ammannia baccifera* were prominent in WSR.

### 3.5. Effect of Crop Management Practices and Weed Management Practices on Grain Yield 

**Figure 3:**
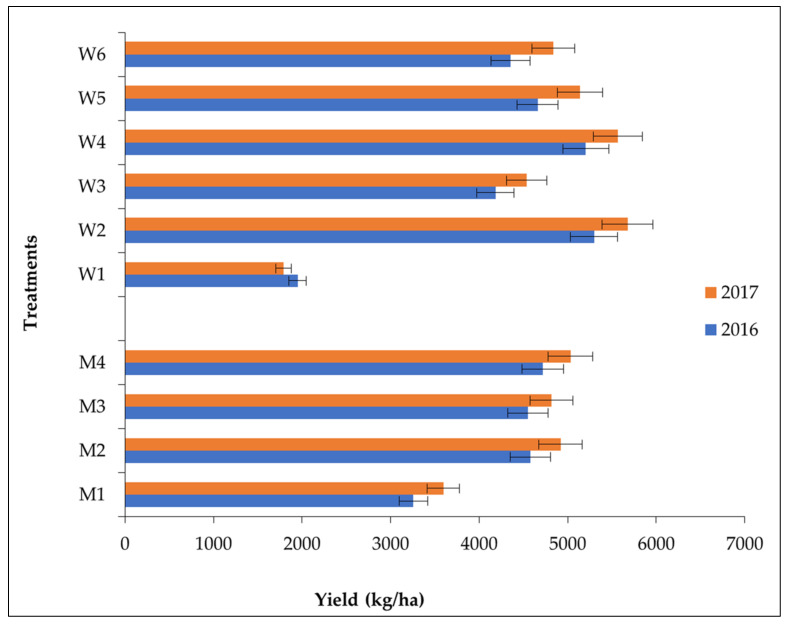
Effect of crop establishment methods and weed management practices on yield of rice.

Among the different crop establishment methods, PTR recorded the highest grain yield (4717 and 5033 kg ha^−1^), followed by WSR (4579 and 4919 kg ha^−1^), NPTR (4549 and 4816 kg ha^−1^), and DSR (3257 and 3595 kg ha^−1^). Among the different weed management practices (Figure 3), application of Bensulfuron methyl 0.6% + Pretilachlor 6% (PE) 0.660 kg ha^−1^+ Hand weeding (HW) at 30 DAS/T recorded the highest grain yield (5298 and 5675 kg ha^−1^, respectively) during both years, followed by the application of Bensulfuron methyl 0.6% + Pretilachlor 6% (PE) 0.495 kg ha^−1^ + Bispyribac-Sodium (POE) 0.025 kg ha^−1^ at 15 DAS/T (5205 and 5565 kg ha^−1^) and CW + HW (4659 and 4839 kg ha^−1^), while the lowest was recorded in weedy check (1951 and 1790 kg ha^−1^, respectively) during both years.

## 4. Discussion

The impact of herbicides on soil microbiological and biochemical activity cannot be described by simple relationships as pesticides may be composed of one or more than one active ingredient that affects the soil microbial properties differently and may be toxic to soil microbes [47].

### 4.1. Effect of Crop Establishment Methods and Weed Management Practices -on Soil Microbial Activity

Significant inhibition of herbicides on microbial population exists that varies with the type of herbicides and dose of herbicide applied. Herbicide application causes a transient impact on microbial growth when applied at a recommended dose. There was an increasing trend of inhibition of microbial growth from initial application to 15 days after application, and no inhibition was found after 15 days after application (DAA) to harvest [48]. There coexists the ability of some microorganisms to degrade the herbicide, while some others are affected adversely by the type of herbicide and rate of application [49]. This has also been confirmed by the results of Zain et al. [50] who opined that the impact of herbicide may be stimulating or depressive on the growth of microorganisms, depending on the chemical types and dose, microbial species, and environmental factors. Microorganisms can degrade herbicides and utilize the products as a source of biogenic elements for their physiological activity, however, the herbicides have a toxic effect on microorganisms, reducing their abundance, activity, and diversity; while the toxic effect of herbicides is more severe immediately after application, at a later stage, microorganisms take part in the herbicides degradation process, converting it into carbon-rich substrates that in turn maximizes the microbial population in the rhizosphere [51].

Higher microbial activity (bacteria, fungi, and actinomycetes) was recorded under unpuddled transplanted rice conditions. While an initial decline in microbial activity was recorded immediately after both pre- and post-emergence herbicide application, that increased afterward, the rate of decline was dose-dependent, where a higher decrease was recorded with a higher concentration of herbicides. Similar results were also observed by Latha and Gopal [34] and Ramalakshmi et al. [35]. A decrease in microbial population was highest after the initial pre-emergence application of Bensulfuron methyl 0.6% + Pretilachlor 6% (PE) at 0.660 kg ha^−1^, followed by application of Bensulfuron methyl 0.6% + Pretilachlor 6% (PE) at 0.495 kg ha^−1^, while a continuous increase in microbial population was recorded where there were no chemical treatments applied. After the initial decline, there was an increase in microbial population unless there was no further additional chemical application. These results are also supported by Chauhan et al. [52] who revealed that the initial decrease followed by an increase in microbial population could also be due to microbial multiplication on the increased supply of nutrients available by the herbicide’s degradation. The highest increase in microbial population and enzymatic activity was noticed in brown/green manuring plots. Jilani et al. [53] revealed that the organic amendments hold great promise as a source of multiple nutrients and the ability to improve soil characteristics.

### 4.2. Effect of Crop Establishment Methods and Weed Management Practices on Soil Enzymatic Activity

The activities of the soil enzymes urease, phosphatase, and dehydrogenase, as affected by different herbicides and weed management practices, have been discussed due to the significant role of these soil enzymes on soil biochemistry, nutrient availability, and plant growth. The urease enzyme catalyzes urea hydrolysis, which is susceptible to different soil-applied herbicides. It is a very useful soil quality indicator that is also widely used to analyze the xenobiotics’ effect on different metabolic activities in the soil [54]. Higher urease activity was recorded under puddled transplanted conditions, followed by wet seeded rice, un-puddled transplanted rice, and dry seeded rice, where urease activity was higher under the flooded condition with a declining trend with the unflooded, dry condition among various weed management practices. Increasing activity of urease with the application of herbicides such as Alachlor, Butachlor, Propaquizafop, and Imazethapyr was recorded under flooded conditions [55,56]. From the study on herbicide, it was recorded that a higher dose of application of Bensulfuron methyl 0.6% + Pretilachlor 6% (PE) at 0.660 kg ha^−1^ recorded a greater decrease in initial urease activity than application at a dose of Bensulfuron methyl 0.6% + Pretilachlor 6% (PE) at 0.495 kg ha^−1^, however, under both cases, the urease activity increased on a later stage, which was little higher at higher dose, that may be due to the degradation of the herbicide to biocarbon by microorganisms and its utilization that in turn may have increased the activity of soil urease, while again with the application of bispyribac sodium at 15 DAS/T at 0.025 kg ha^−1^ decreased the urease activity recorded at 21 DAS/T, that again increased at 28 DAS/T. A decrease in soil urease activity by increasing the concentration of various herbicides has also been reported by various authors [57,58,59]. Some authors have also found no effect of herbicides such as 2,4-dichlorofenoxy acetic acid, butachlor, and oxyfluorfen on soil urease activity [60].

The soil enzyme phosphatase plays an important role in converting organically bound phosphorous to an inorganic form, making it available for soil microorganisms as well as for plants. Both the stimulatory and inhibitory impact of herbicides on soil phosphatase activity has been reported by various researchers [54,61,62]. Higher activity of phosphatase was recorded in unpuddled transplanted conditions, while the lowest trend was recorded in puddled transplanted conditions. A similar report has been recorded by Rasool et al. [56] from their study on butachlor, which was also found to have an inhibitory impact on phosphatase activity under flooded conditions, while its impact was stimulatory under unflooded conditions. A steady increase in phosphatase activity was recorded in brown/green manured treatment and with the application of cono weeding and hand weeding treatments; better soil aeration and/or higher root activity may be the cause behind higher microbial and phosphatase activity, while the application of the pre-emergence herbicide Bensulfuron methyl 0.6% + Pretilachlor 6% (PE) at a higher dose of 0.660 kg ha^−1^ recorded a higher decrease in phosphatase activity at a dose of 0.495 kg ha^−1^, while the increase in enzyme activity at a later stage was also higher where a higher dose of chemicals was applied (W_2_). The stimulatory impact of the herbicide fomesafen has also been reported by Vladoiu et al. [63], Filimon et al. [64], and Ba’cmaga et al. [54], while the inhibitory effect of herbicides on phosphatase activity has been recorded by Filimon et al. [61] and Muñoz-Leoz et al. [64].

An activity study of the soil enzyme dehydrogenase has been considered one of the important methods of determining the effect of various chemical herbicides and pesticides on soil biochemistry. In the present study, an increase in dehydrogenase activity was recorded under brown/green manuring, which may be due to higher root activity, that may have increased the microbial population in various soil conditions, while at 7 DAS/T, a dose-dependent decline in dehydrogenase activity was recorded due to the pre-emergence application of herbicide Bensulfuron methyl 0.6% + Pretilachlor 6% (PE), that later increased at continuously after 14 DAS/T, and again, with the application of post-emergence herbicide bispyribac sodium, a declining trend was recorded in W_4_ which increased at 28 DAS/T. A dose-dependent decrease in dehydrogenase activity due to herbicide S-metolachlor has also been reported by Filimon et al. [61] and Rasool et al. [56] whose studies found a detrimental effect of the herbicide butachlor on dehydrogenase activity under an unflooded soil condition while the impact was stimulatory under a flooded condition.

### 4.3. Effect of Crop Establishment Methods and Weed Management Practices on Weed Count and Population

It is imperative to know the weeds associated with any crop before recommending a control measure. Weeds present in any crop are mostly regulated by the growing season, cultural factors adopted by the farmer, and agro-ecological factors. A thorough study of weed taxonomy and biology is essential for success and efficient weed management. A critical study from the commencement of the crop (rice) until harvest indicated that as many as 15 different types of weeds existed in the crop field. Major weed flora in the experimental site enlisted of grasses, viz., *Echinochloa colona* (L.) Link, *Echinochloa crusgalli* (L.) Beauv, *Leptochloa chinensis* (L.) Nees, *Paspalum distichum* (L.), Sedges, *Cyperus iria* (L.), *Cyperus difformis* (L.), *Fimbristylis miliacea* (L.) Vahl, broad leave weeds, viz., *Eclipta alba* (L.) Hassk, *Alternanthera philoxeroids* (Mart.) Griseb, *Aeschynomene indica* (L.), *Ammannia baccifera* (L), *Cyanotis cucullata* (Roth) Kunth, *Spenoclea zeylanica*, *Spilanthes acmella* (L.), and *Ludwigia parviflora* (L.). Similar weed species in rice have been reported by Mahajan et al. [65] and Mohanty et al. [66].

### 4.4. Effect of Crop Establishment Methods and Weed Management Practices on Grain Yield of Rice

Higher yield in PTR might be due to higher yield attributing characters which may be the ultimate result of better availability and utilization of nutrients in properly spaced transplanted rice during the panicle growth period [67]. Similar results of higher yield were also observed by Jaiswal and Singh [68], Chauhan et al. [69], and Saharawat et al. [70] who recorded 0.45–0.61 t ha^−1^ lesser yield in both dry and wet direct-seeded rice than transplanted rice. Higher yield with the application of Bensulfuron methyl 0.6% + Pretilachlor 6% (PE) at 0.66 kg ha^−1^ + HW at 30 DAS/T might be the combined result of higher yield attributing characters and the lowest crop weed completion due to better weed control throughout the crop growth seasons, effective utilization of moisture, nutrients, light, and space as a whole. Similar results have also been observed by Sunil et al. [71], Reddy et al. [72], and Kumar et al. [73].

## 5. Conclusions

The present study revealed that microbial activity varies with different crop establishment methods and different weed management practices. A better microbial population with the microbial activity of dehydrogenase and phosphatase was recorded after an initial decline due to herbicide application in unpuddled transplanted conditions, providing a suitable environment for soil activity and soil health. The application of Bensulfuron methyl 0.6% + Pretilachlor 6% (PE) at 0.660 kg ha^−1^ recorded a greater decrease in initial urease, phosphatase, and dehydrogenase activity than the application of a dose of Bensulfuron methyl 0.6% + Pretilachlor 6% (PE) at 0.495 kg ha^−1^. However, in both cases, the urease activity increased at a later stage, which was a little more than at a higher dose of herbicide, which might have increased the degradation of the herbicide to biocarbon by microorganisms, and its utilization, in turn, may have increased the activity of soil enzymes. Higher and better microbial activity with increased activity with crop duration was recorded in treatments where weed management was organically done. In treatments with chemical weed control, microbial activity suffered immediately after application but regained with time and the stage of the crop. Further study of different and new herbicides and their effect on soil microbial activity, soil enzymes, and bioassays will help the scientific community gain a better understanding of soil health.

## Figures and Tables

**Table 1:** Textural class of soil of the experimental area.

Sl. No	Constituents	0–10 cm	Method Followed
1	Sand (%)	83.7	Bouycous Hydrometer method [37]
2	Silt (%)	6.8	
3	Clay (%)	10.4	
4	Textural classes	Sandy loam	

**Table 2:** Chemical composition of the soil.

Parameters	0–10 cm		Method Adopted
	Values	Remarks	
pH	5.9	Acidic	Digital electronic pH meter with 1:2.5, soil: water [38]
Organic carbon (%)	0.53	Medium	Walkely and Black’s rapid titration method [38]
Available Nitrogen (kg ha^−1^)	226.4	Low	Alkaline potassium permanganate method [39]
Available Phosphorus (kg ha^−1^)	32.6	High	Bray’s-1 method [38]
Available Potassium (kg ha^−1^)	132.6	Medium	Ammonium acetate flame photometer method [38]

**Table 3:** Initial microbiological properties of experimental soil.

Parameters	Values	Methods
Total bacterial population	41.9 (×10^6^ CFU/g soil)	Serial dilution and spread plate technique [40]
Total fungal population	11.9 (×10^4^ CFU/g soil)	Serial dilution and spread plate technique [40]
Total actinomycetes population	34.2 (×10^3^ CFU/g soil)	Serial dilution and spread plate technique [40]
Urease	26.7 (μg NH3 released/g soil/h)	Tabatabai [41]
Alkaline phosphatase	178.5 (μg p-nitrophenol/g soil/h)	Tabatabai and Bremner [42]
Dehydrogenase	84.1 (μg TPF/g soil/24 h)	Tabatabai [41]

**Table 4:** Treatment details of the experiment.

Main Plots	Crop Establishment Methods
M_1_	Direct Seeded Rice (DSR)
M_2_	Wet seeded Rice (WSR)
M_3_	Non-Puddled Transplanted Rice (NPTR)
M_4_	Puddled Transplanted Rice (PTR)
**Sub Plots**	**Weed Management Practices**
W_1_	Weedy Check
W_2_	Bensulfuron methyl 0.6% + Pretilachlor 6% (PE) 0.660 kg ha^−1^ + Hand weeding (HW) at 30 DAS/T
W_3_	Bensulfuron methyl 0.6% + Pretilachlor 6% (PE) 0.495 kg ha^−1^ + HW at 30 DAS/T
W_4_	Bensulfuron methyl 0.6% + Pretilachlor 6% (PE) 0.495 kg ha^−1^ + Bispyribac-Sodium (POE) 0.025 kg ha^−1^ at 15 DAS/T,
W_5_	Cono weeding at 15 DAS/T + hand weeding 30 DAS/T
W_6_	Brown manuring/Green manuring

**Table 5:** Total bacterial population (×10^6^ CFU g^−1^ soil) in rice soil as influenced by crop establishment methods and weed management practices.

Total Bacterial Population (×10^6^ CFU g^−1^ Soil)								
Treatments	7 DAS/T		14 DAS/T		21 DAS/T		28 DAS/T	
	2016	2017	2016	2017	2016	2017	2016	2017
Establishment Methods								
M_1_	37.57 ^c^	38.48 ^c^	40.83 ^c^	41.75 ^c^	42.97 ^c^	43.73 ^c^	44.68 ^b^	46.40 ^b^
M_2_	38.53 ^b^	39.38 ^b^	41.87 ^b^	43.33 ^b^	44.08 ^b^	44.70 ^b^	45.33 ^b^	45.47 ^b^
M_3_	39.47 ^a^	40.50 ^a^	43.82 ^a^	44.18 ^a^	45.67 ^a^	45.83 ^a^	49.57 ^a^	48.85 ^a^
M_4_	35.93 ^d^	37.78 ^d^	40.65 ^c^	40.88 ^d^	43.08 ^c^	42.03 ^d^	45.32 ^b^	46.45 ^ab^
SEm (±)	0.081	0.063	0.067	0.064	0.049	0.066	0.199	0.348
CD (0.05)	0.282	0.218	0.23	0.221	0.17	0.23	0.688	1.204
Weed Management Practice								
W_1_	42.00 ^b^	43.48 ^a^	42.93 ^c^	44.58 ^c^	43.88 ^c^	45.98 ^c^	44.23 ^c^	47.50 ^b^
W_2_	31.93 ^d^	32.93 ^c^	38.53 ^d^	40.38 ^d^	42.78 ^d^	45.45 ^d^	44.95 ^c^	47.93 ^ab^
W_3_	33.25 ^c^	35.30 ^b^	35.83 ^e^	39.40 ^e^	38.35 ^e^	44.68 ^e^	40.75 ^d^	46.53 ^b^
W_4_	33.25 ^c^	35.30 ^b^	35.83 ^e^	39.40 ^e^	33.98 ^f^	32.55 ^f^	38.43 ^e^	41.45 ^c^
W_5_	41.98 ^b^	43.48 ^a^	44.05 ^b^	45.20 ^b^	46.25 ^b^	47.10 ^b^	53.75 ^b^	49.73 ^a^
W_6_	44.85 ^a^	43.75 ^a^	53.60 ^a^	46.28 ^a^	58.48 ^a^	48.70 ^a^	55.25 ^a^	47.63 ^ab^
SEm (±)	0.074	0.067	0.01	0.019	0.014	0.011	0.234	0.428
CD (0.05)	0.215	0.195	0.03	0.056	0.042	0.031	0.684	1.249

M_1_—Direct Seeded Rice (DSR), M_2_— Wet Seeded Rice (WSR), M_3_—Unpuddled Transplanted Rice (NPTR), M_4_—Puddled Transplanted Rice (PTR), W_1_—Weedy check, W_2_—Bensulfuron methyl 0.6% + Pretilachlor 6% (PE) 0.660 kgha^−1^ + Hand weeding (HW) at 30 DAS/T, W_3_—Bensulfuron methyl 0.6% + Pretilachlor 6% (PE) 0.495 kgha^−1^ + HW at 30 DAS/T, W_4_—Bensulfuron methyl 0.6% + Pretilachlor 6% (PE) 0.495 kgha^−1^ + Bispyribac-Sodium (POE) 0.025 kg ha^−1^ at 15 DAS/T, W_5_—Cono weeding at 15 DAS/T + hand weeding 30 DAS/T, W_6_—Brown manuring/Green manuring. SE, standard Error, CD, Critical difference at 5% level of probability. Different superscript letters indicate a significant difference between the mean.

**Table 6:** Total Fungi population (×10^4^ CFU g^−1^ soil) in rice soil as influenced by crop establishment methods and weed management practices.

Total Fungi Population (×10^4^ CFU g^−1^ Soil)								
Treatments	7 DAS/T		14 DAS/T		21 DAS/T		28 DAS/T	
	2016	2017	2016	2017	2016	2017	2016	2017
Establishment Methods								
M_1_	9.95 ^c^	11.43 ^c^	15.08 ^c^	17.95 ^a^	18.38 ^c^	20.22 ^c^	24.35 ^c^	27.38 ^d^
M_2_	10.62 ^b^	12.48 ^a^	16.60 ^b^	20.35 ^a^	19.70 ^b^	22.17 ^b^	25.07 ^b^	28.37 ^b^
M_3_	11.28 ^a^	12.07 ^b^	17.35 ^a^	21.47 ^a^	20.80 ^a^	23.47 ^a^	28.92 ^a^	31.98 ^a^
M_4_	9.87 ^c^	11.62 ^c^	14.82 ^c^	17.13 ^a^	17.22 ^d^	20.08 ^c^	24.98 ^b^	27.92 ^c^
SEm (±)	0.05	0.037	0.042	0.054	0.045	0.037	0.044	0.046
CD (0.05)	0.173	0.127	0.145	1.743	0.156	0.129	0.151	0.16
Weed management practices								
W_1_	12.15 ^b^	13.73 ^c^	15.60 ^c^	18.28 ^a^	19.65 ^d^	20.10 ^d^	24.40 ^d^	27.88 ^c^
W_2_	7.63 ^e^	8.58 ^e^	16.45 ^b^	20.20 ^a^	21.90 ^b^	23.95 ^b^	27.75 ^a^	30.63 ^a^
W_3_	9.05 ^d^	9.88 ^d^	14.23 ^d^	18.13 ^a^	17.90 ^e^	22.38 ^c^	26.88 ^b^	29.70 ^b^
W_4_	9.05 ^d^	9.88 ^d^	14.23 ^d^	18.23 ^a^	9.88 ^f^	12.43 ^e^	22.10 ^e^	27.18 ^d^
W_5_	12.05 ^c^	13.90 ^b^	15.60 ^c^	18.68 ^a^	20.05 ^c^	22.40 ^c^	28.00 ^a^	30.40 ^a^
W_6_	12.65 a	15.45 a	19.68 ^a^	21.85 ^a^	24.78 ^a^	27.65 ^a^	25.85 ^c^	27.70 ^c^
SEm (±)	0.006	0.015	0.007	0.598	0.011	0.016	0.059	0.041
CD (0.05)	0.018	0.044	0.02	1.746	0.033	0.47	0.171	0.121

Table 4 may be referred to for treatment details. SE, standard Error, CD, Critical difference at 5% probability level. Different superscript letters indicate a significant difference between the mean.

**Table 7:** Total Actinomycetes population (×10^3^ CFU g^−1^ soil) in rice soil as influenced by crop establishment methods and weed management practices.

Total Actinomycetes Population (×10^3^ CFU g^−1^ Soil)								
Treatments	7 DAS/T		14 DAS/T		21 DAS/T		28 DAS/T	
	2016	2017	2016	2017	2016	2017	2016	2017
Establishment Methods								
M_1_	31.97 ^c^	32.59 ^a^	36.22 ^c^	36.97 ^c^	39.73 ^c^	41.02 ^b^	44.20 ^c^	43.81 ^b^
M_2_	32.55 ^b^	33.35 ^a^	38.13 ^b^	38.15 ^b^	41.13 ^b^	42.23 ^a^	45.23 ^b^	44.87 ^a^
M_3_	34.83 ^a^	34.38 ^a^	39.55 a	39.15 ^a^	42.65 ^a^	42.45 ^a^	48.22 ^a^	45.23 ^a^
M_4_	31.17 ^d^	31.68 ^a^	34.78 ^d^	36.27 ^d^	38.02 ^d^	39.60 ^c^	43.42 ^d^	42.75 ^c^
SEm (±)	0.056	0.972	0.044	0.051	0.046	0.028	0.075	0.051
CD (0.05)	0.195	NS	0.153	0.178	0.159	0.096	0.261	0.176
Weed Management Practices								
W_1_	38.30 ^b^	36.48 ^a^	40.23 ^c^	38.38 ^c^	42.33 ^c^	42.10 ^d^	46.05 ^d^	43.85 ^d^
W_2_	24.55 ^d^	26.84 ^b^	33.00 ^d^	34.83 ^d^	41.88 ^d^	42.35 ^c^	47.58 ^b^	45.95 ^b^
W_3_	27.03 ^c^	28.78 ^b^	31.90 ^e^	33.58 ^e^	39.23 ^e^	40.88 ^e^	45.08 ^e^	45.03 c
W_4_	27.03 ^c^	28.78 ^b^	31.90 ^e^	33.58 ^e^	24.60 ^f^	29.33 ^f^	35.88 ^f^	39.53 ^f^
W_5_	38.30 ^b^	37.98 ^a^	41.38 ^b^	41.93 ^b^	46.20 ^b^	45.95 ^b^	50.00 ^a^	49.35 ^a^
W_6_	40.58 ^a^	39.18 ^a^	44.63 ^a^	43.53 ^a^	48.08 ^a^	47.35 ^a^	47.03 ^c^	41.29 ^e^
SEm (±)	0.015	0.745	0.017	0.01	0.019	0.017	0.066	0.06
CD (0.05)	0.043	2.174	0.05	0.03	0.054	0.049	0.0192	0.174

For treatment details, Table 4 may be referred to. SE, standard Error, CD, Critical difference at 5% probability level. Different superscript letters indicate a significant difference between the mean.

**Table 8:** Crop establishment methods and weed management practices influence the activity of the urease enzyme (μg NH3 released g^−1^ soil h^−1^) in rice soil.

The Activity of Soil Enzyme Urease (μg NH3 Released g^−1^ Soil hr^−1^)								
Treatments	7 DAS/T		14 DAS/T		21 DAS/T		28 DAS/T	
	2016	2017	2016	2017	2016	2017	2016	2017
Establishment Methods								
M_1_	21.62 ^c^	23.57 ^d^	23.77 ^b^	26.20 ^d^	24.93 ^b^	28.05 ^b^	27.60 ^c^	31.50 ^c^
M_2_	23.60 ^b^	25.58 ^b^	25.75 ^a^	29.28 ^b^	26.78 ^b^	30.28 ^a^	28.28 ^b^	33.08 ^b^
M_3_	23.05 ^b^	25.03 ^c^	24.43 ^b^	28.17 ^c^	25.73 ^b^	30.30 ^a^	26.78 ^d^	31.07 ^d^
M_4_	24.57 ^a^	27.00 ^a^	26.75 ^a^	30.58 ^a^	27.44 ^a^	31.36 ^a^	30.53 ^a^	35.02 ^a^
SEm (±)	0.481	0.039	0.118	0.118	0.341	0.185	0.045	0.049
CD (0.05)	1.664	0.136	0.408	0.407	1.181	0.639	0.156	0.153
Weed Management Practices								
W_1_	26.95 ^a^	30.55 ^a^	22.35 ^c^	31.68 ^a^	21.30 ^d^	32.78 ^b^	20.83 ^f^	33.38 ^d^
W_2_	19.08 ^b^	19.33 ^d^	25.50 ^b^	26.58 ^b^	28.45 ^b^	29.25 ^c^	29.90 ^c^	33.48 ^c^
W_3_	19.45 ^b^	20.55 ^c^	23.15 ^c^	24.30 ^c^	25.93 ^c^	27.35 ^d^	28.00 ^d^	31.55 ^e^
W_4_	19.50 ^b^	20.55 ^c^	23.15 ^c^	24.30 ^c^	17.92 ^e^	22.23 ^e^	25.55 ^e^	27.73 ^f^
W_5_	27.13 ^a^	30.30 ^b^	28.10 ^a^	31.90 ^a^	30.48 ^b^	33.89 ^b^	32.23 ^b^	35.78 ^a^
W_6_	27.15 ^a^	30.50 ^a^	28.80 ^a^	32.60 ^a^	33.28 ^a^	34.49 ^a^	33.30 ^a^	34.10 ^b^
SEm (±)	0.592	0.023	0.155	0.173	0.434	0.197	0.013	0.016
CD (0.05)	1.728	0.066	0.453	0.505	1.267	0.575	0.037	0.045

For treatment details, Table 4 may be referred to. SE, standard Error, CD, Critical difference at 5% probability level. Different superscript letters indicate a significant difference between the mean.

**Table 9:** The activity of soil enzyme alkaline phosphatase in rice as influenced by crop establishment methods and weed management practices.

The Activity of Soil Enzyme Alkaline Phosphatase (μg p-nitro-phenol g^−1^ soil h^−1^)								
Treatments	7 DAS/T		14 DAS/T		21 DAS/T		28 DAS/T	
	2016	2017	2016	2017	2016	2017	2016	2017
Establishment Methods								
M_1_	155.45 ^c^	166.33 ^b^	175.45 ^b^	175.35 ^bc^	180.95 ^c^	183.28 ^bc^	187.10 ^b^	194.62 ^c^
M_2_	163.05 ^b^	169.05 ^b^	179.93 ^ab^	179.53 ^b^	185.90 ^b^	188.55 ^b^	190.45 ^b^	201.07 ^b^
M_3_	171.32 ^a^	175.68 ^a^	186.28 ^a^	191.70 ^a^	192.17 ^a^	197.42 ^a^	197.95 ^a^	215.47 ^a^
M_4_	161.52 ^b^	165.13 ^b^	174.00 ^b^	171.32 ^c^	180.17 ^c^	178.67 ^c^	189.95 ^b^	189.08 ^d^
SEm (±)	0.413	0.565	0.8	0.696	0.286	0.872	0.45	0.506
CD (0.05)	1.427	1.953	2.767	2.407	0.99	3.019	1.556	1.751
Weed Management Practices								
W_1_	180.70 ^a^	183.25 ^c^	184.70 ^b^	190.50 ^c^	164.83 ^e^	192.05 ^c^	160.23 ^e^	194.08 ^c^
W_2_	141.83 ^e^	146.58 ^e^	168.40 ^c^	162.95 ^d^	184.63 ^c^	185.68 ^c^	189.05 ^b^	200.50 ^b^
W_3_	148.70 ^d^	152.70 ^d^	160.03 ^d^	158.93 ^d^	176.38 ^d^	172.85 ^d^	184.20 ^c^	193.00 ^c^
W_4_	148.70 ^d^	152.70 ^d^	160.03 ^d^	158.93 ^d^	149.10 ^f^	149.05 ^e^	178.65 ^d^	181.33 ^d^
W_5_	179.53 ^b^	187.85 ^b^	188.40 ^b^	197.13 ^b^	209.40 ^b^	203.60 ^b^	216.70 ^a^	214.63 ^a^
W_6_	177.55 ^c^	191.23 ^a^	211.95 ^a^	208.43 ^a^	224.45 ^a^	218.65 ^a^	219.35 ^a^	216.83 ^a^
SEm (±)	0.104	0.227	0.66	1.132	0.39	1.335	0.707	1.118
CD (0.05)	0.304	0.662	1.926	3.304	1.138	3.896	2.063	3.262

For treatment details, Table 4 may be referred to. SE, standard Error, CD, Critical difference at 5% probability level. Different superscript letters indicate a significant difference between the mean.

**Table 10:** The activity of soil enzyme dehydrogenase in rice as influenced by crop establishment methods and weed management practices.

The Activity of Soil Enzyme Dehydrogenase (μg TPF g^−1^ soil 24 h^−1^)								
Treatments	7DAS		14DAS		21DAS		28DAS	
	2016	2017	2016	2017	2016	2017	2016	2017
Establishment Methods								
M_1_	80.75 ^b^	81.13 ^b^	88.67 ^b^	92.05 ^b^	91.63 ^b^	99.45 ^b^	94.22 c	101.52 ^c^
M_2_	82.67 ^ab^	81.63 ^ab^	90.83 ^ab^	92.25 ^b^	94.32 ^b^	99.95 ^b^	97.20 ^b^	105.17 ^bc^
M_3_	84.43 ^a^	86.07 ^a^	94.32 ^a^	96.03 ^a^	98.92 ^a^	107.95 ^a^	103.65 ^a^	112.95 ^a^
M_4_	80.07 ^b^	82.00 ^ab^	89.08 ^b^	91.62 ^b^	92.15 ^b^	99.80 ^b^	97.13 ^b^	106.50 ^b^
Sem (±)	0.365	0.529	0.491	0.307	0.334	0.339	0.299	0.462
CD (0.05)	1.261	1.831	1.698	1.061	1.154	1.174	1.034	1.601
Weed Management Practices								
W_1_	86.63 ^b^	88.20 ^b^	80.70 ^d^	92.50 ^c^	74.00 ^e^	99.45 ^d^	69.00 ^d^	104.28 ^d^
W_2_	72.55 ^c^	71.95 ^c^	93.78 ^b^	89.88 ^d^	106.23 ^b^	109.50 ^b^	108.38 ^a^	113.25 ^b^
W_3_	74.28 ^c^	74.73 ^c^	83.68 ^c^	87.08 ^e^	92.15 ^d^	102.73 ^c^	103.68 ^b^	107.50 ^c^
W_4_	74.28 ^c^	74.73 ^c^	83.68 c	87.08 ^e^	75.48 ^e^	75.00 ^e^	92.30 ^c^	90.48 ^e^
W_5_	86.63 ^b^	88.65 ^b^	94.55 ^b^	95.98 ^b^	102.18 ^c^	109.53 ^b^	106.70 ^a^	115.60 ^a^
W_6_	97.53 ^a^	98.00 ^a^	107.98 ^a^	105.43 ^a^	115.50 ^a^	114.53 ^a^	108.25 ^a^	108.10 ^c^
SEm (±)	0.356	0.496	0.422	0.323	0.454	0.495	0.337	0.409
CD (0.05)	1.038	1.446	1.232	0.943	1.326	1.445	0.983	1.196

For treatment details, Table 4 may be referred to. SE, standard Error, CD, Critical difference at 5% probability level. Different superscript letters indicate a significant difference between the mean.

**Table 11:** Multiple correlations between micro-organisms and soil enzymes at different stages were recorded at 7 and 14 DAS in both years.

7 DAS/T in the Year 2016					
Parameters	Bacteria	Fungi	Actinomycetes	Urease	Phosphatase
	1	2	3	4	5
Fungi	0.98 **				
Actinomycetes	0.99 **	0.99 **			
Urease	0.92 **	0.92 **	0.94 **		
Phosphatase	0.95 **	0.98 **	0.97 **	0.95 **	
Dehydrogenase	0.97 **	0.93 **	0.96 **	0.88 **	0.88 **
7 DAS/T in the Year 2017					
Parameters	Bacteria	Fungi	Actinomycetes	Urease	Phosphatase
	1	2	3	4	5
Fungi	0.97 **				
Actinomycetes	0.99 **	0.99 **			
Urease	0.95 **	0.95 **	0.94 **		
Phosphatase	0.99 **	0.98 **	1.00 **	0.95 **	
Dehydrogenase	0.94 **	0.97 **	0.97 **	0.91 **	0.97 **
14 DAS/T in the Year 2016					
Parameters	Bacteria	Fungi	Actinomycetes	Urease	Phosphatase
	1	2	3	4	5
Fungi	0.88 **				
Actinomycetes	0.94 **	0.76 **			
Urease	0.67 *	0.59 *	0.55 *		
Phosphatase	0.99 **	0.86 **	0.97 **	0.65 *	
Dehydrogenase	0.80 **	0.87 **	0.63 *	0.84 **	0.77 **
14 DAS/T in the Year 2017					
Parameters	Bacteria	Fungi	Actinomycetes	Urease	Phosphatase
	1	2	3	4	5
Fungi	0.55				
Actinomycetes	0.97 **	0.53			
Urease	0.85 **	0.25	0.85 **		
Phosphatase	0.99 **	0.53	0.98 **	0.85 **	
Dehydrogenase	0.87 **	0.66*	0.94 **	0.75 **	0.92 **

* Correlation is significant at a 5% level of probability (1-tailed); ** Correlation is significant at a 1% level of probability (1-tailed).

**Table 12:** Multiple correlations between micro-organisms and soil enzymes at different stages were recorded at 21 and 28 DAS/T in both years.

21 DAS/T in the Year 2016					
Parameters	Bacteria	Fungi	Actinomycetes	Urease	Phosphatase
	1	2	3	4	5
Fungi	0.85 **				
Actinomycetes	0.82 **	0.95 **			
Urease	0.79 **	0.81 **	0.81 **		
Phosphatase	0.89 **	0.82 **	0.84 **	0.94 **	
Dehydrogenase	0.73 **	0.77 **	0.69 *	0.93 **	0.90 **
21 DAS/T in the year 2017					
Parameters	Bacteria	Fungi	Actinomycetes	Urease	Phosphatase
	1	2	3	4	5
Fungi	0.92 **				
Actinomycetes	0.98 **	0.92 **			
Urease	0.86 **	0.75 **	0.89 **		
Phosphatase	0.90 **	0.87 **	0.94 **	0.91 **	
Dehydrogenase	0.95 **	0.97 **	0.96 **	0.81 **	0.88 **
28 DAS/T in the year 2016					
Parameters	Bacteria	Fungi	Actinomycetes	Urease	Phosphatase
	1	2	3	4	5
Fungi	0.55 *				
Actinomycetes	0.76 **	0.90 **			
Urease	0.62 *	0.40	0.60 *		
Phosphatase	0.83 **	0.51	0.68 *	0.90 **	
Dehydrogenase	0.43	0.60 *	0.80 **	0.86 **	0.81 **
28 DAS/T in the year 2017					
Parameters	Bacteria	Fungi	Actinomycetes	Urease	Phosphatase
	1	2	3	4	5
Fungi	0.65 *				
Actinomycetes	0.75 **	0.71 *			
Urease	0.73 *	0.16	0.55 *		
Phosphatase	0.78 **	0.56 *	0.50	0.48	
Dehydrogenase	0.94 **	0.77 **	0.80 **	0.72 **	0.76 **

* Correlation is significant at a 5% level of probability (1-tailed); ** Correlation is significant at a 1% level of probability (1-tailed).

## Data Availability

Data will be available after requesting to corresponding author.
